# Supplementary material for: Anti-MRSA Sesquiterpenes from the Semi-Mangrove Plant Myoporum bontioides A. Gray
Source: Mar Drugs. 2018 Nov 8;16(11):438. doi: 10.3390/md16110438 (PMC6265800; doi:10.3390/md16110438)
Supplement: Supplementary file 1 [file marinedrugs-16-00438-s001.pdf]

Supporting Materials

Anti-MRSA Sesquiterpenes from the Semi-Mangrove Plant *Myoporum Bontioides* A. Gray

Li-Mei Dong [1,†], Li-Lan Huang [2,†], Hang Dai [2], Qiao-Lin Xu [3], Jin-Kui Ouyang [1], Xu-Chao Jia [4], Wen-Xiang Gu [2,*] and Jian-Wen Tan [1,*]

1 State Key Laboratory for Conservation and Utilization of Subtropical Agro-bioresources/Guangdong Key Laboratory for Innovative Development and Utilization of Forest Plant Germplasm, College of Forestry and Landscape Architecture, South China Agricultural University, Guangzhou 510642, China; lmdong@scau.edu.cn (L.-M.D.); ouyangjack@scau.edu.cn (J.-K.O.)

2 College of Materials and Energy, South China Agricultural University, Guangzhou 510642, China; lnhuang3233@163.com (L.-L.H.); hangdai0101@163.com (H.D.)

3 Guangdong Provincial Key Laboratory of Bio-control for the Forest Disease and Pest, Guangdong Academy of Forestry, Guangzhou 510520, China; qlxu@sinogaf.cn

4 Key Laboratory of Functional Foods, Ministry of Agriculture/Guangdong Key Laboratory of Agricultural Products Processing /Sericultural & Agri-Food Research Institute, Guangdong Academy of Agricultural Sciences, Guangzhou 510610, China; jiaxuchao@gdaas.cn (X.-C.J.)

* Correspondence: wenxgu@scau.edu.cn (W.-X.G.); jwtan@scau.edu.cn (J.-W.T.)

† These authors contributed equally to this work.

List of Contents

Figure S1. ^{1}H NMR spectrum of compound **1** in $CDCl_3$.

Figure S2. ^{13}C NMR spectrum of compound **1** in $CDCl_3$.

Figure S3. HSQC spectrum of compound **1** in $CDCl_3$.

Figure S4. ^{1}H-^{1}H COSY spectrum of compound **1** in $CDCl_3$.

Figure S5. HMBC spectrum of compound **1** in $CDCl_3$.

Figure S6. NOESY spectrum of compound **1** in $CDCl_3$.

Figure S7. HRESIMS spectrum of compound **1**.

Figure S8. ^{1}H NMR spectrum of compound **2** in $CDCl_3$.

Figure S9. ^{13}C NMR spectrum of compound **2** in $CDCl_3$.

Figure S10. HSQC spectrum of compound **2** in $CDCl_3$.

Figure S11. ^{1}H-^{1}H COSY spectrum of compound **2** in $CDCl_3$.

Figure S12. HMBC spectrum of compound **2** in $CDCl_3$.

Figure S13. HRESIMS spectrum of compound **2**.

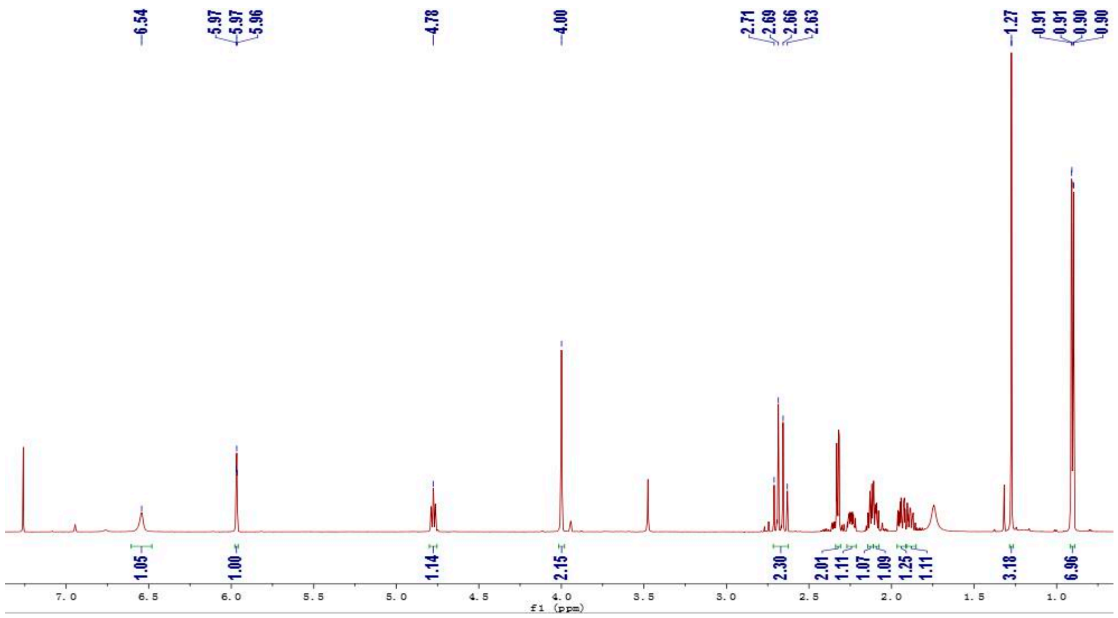

Figure S1. ^{1}H NMR spectrum of compound **1** in $CDCl_3$.

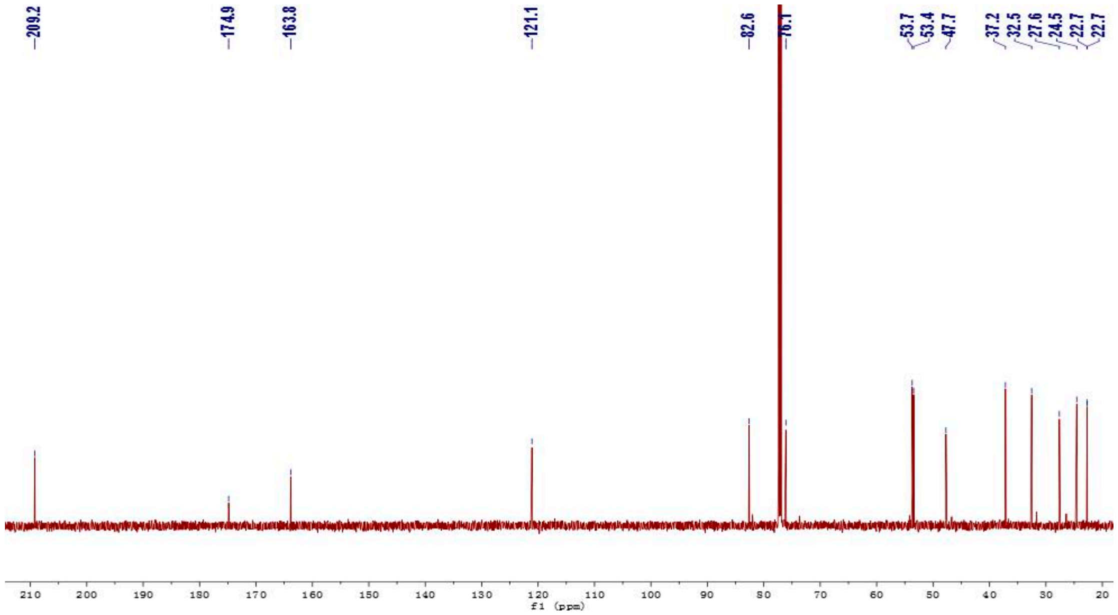

Figure S2. ^{13}C NMR spectrum of compound **1** in $CDCl_3$.

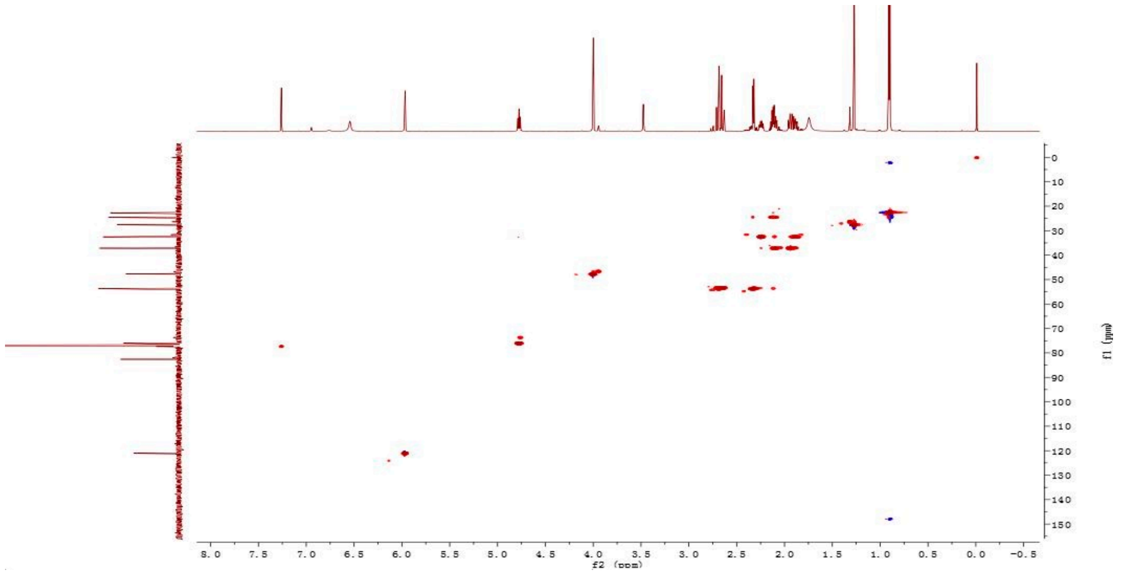

Figure S3. HSQC spectrum of compound **1** in $CDCl_3$.

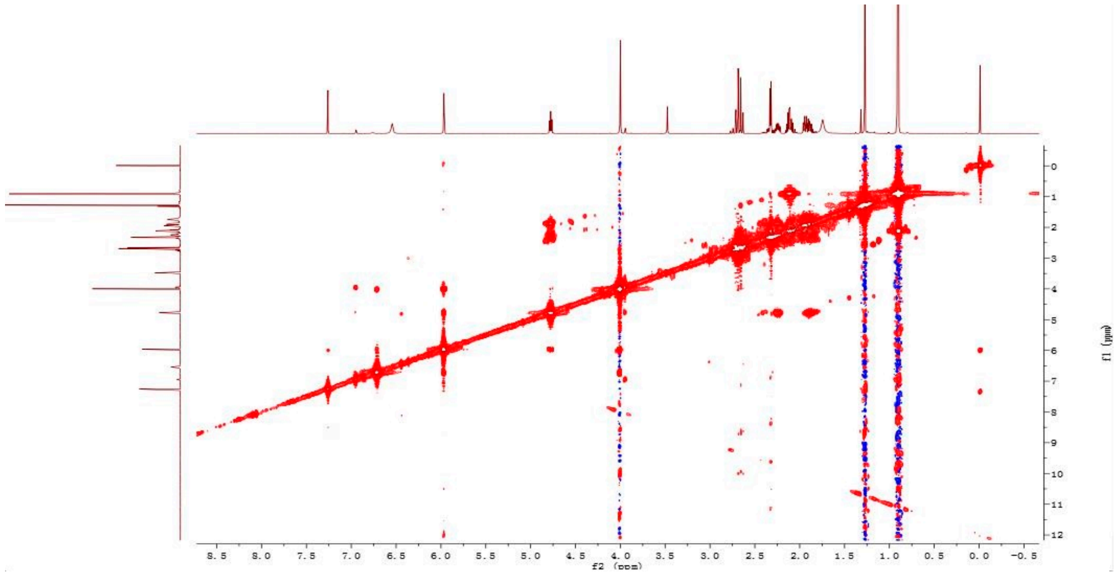

Figure S4. ^1H-^1H COSY spectrum of compound **1** in $CDCl_3$.

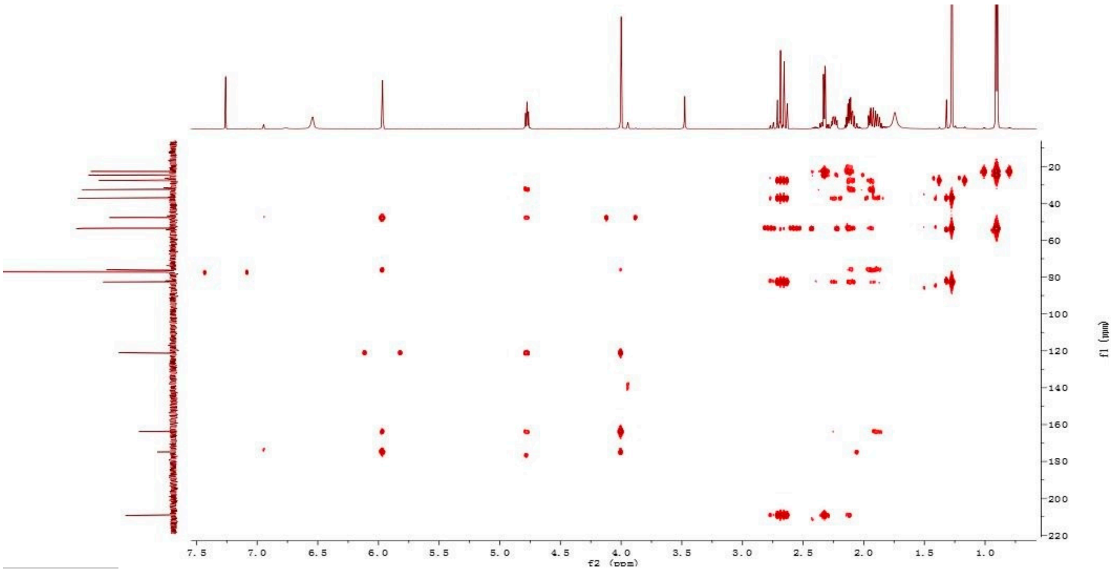

Figure S5. HMBC spectrum of compound **1** in CDCl3.

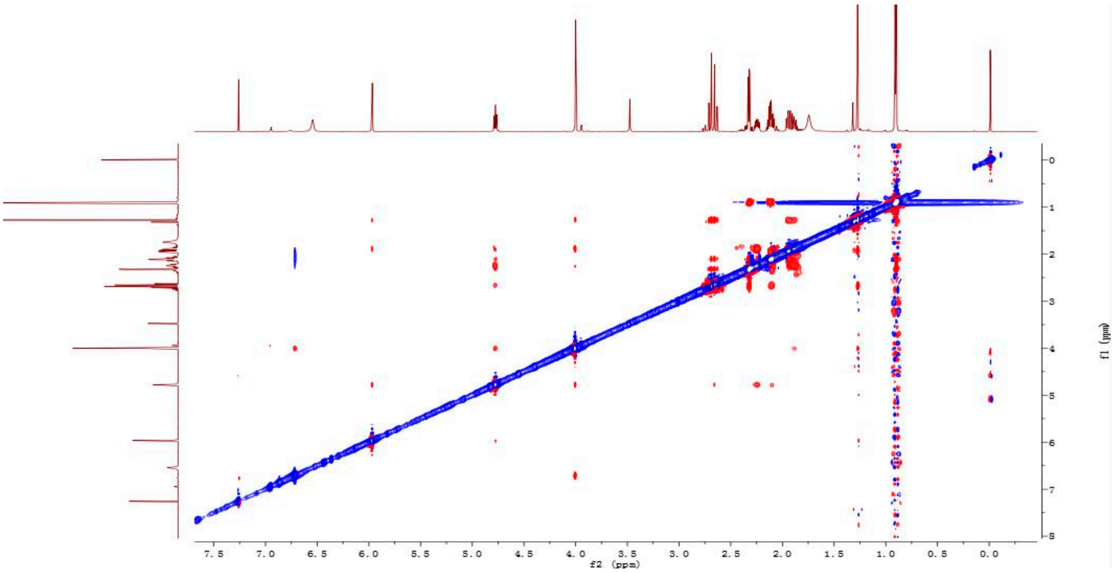

Figure S6. NOESY spectrum of compound **1** in CDCl3.

Mass Spectrum SmartFormula Report

Analysis Info

Analysis Name	D:\Data\MS\data\MB-23_pos.d	Operator	SCSIO
Method	LC_Direct Infusion_pos_100-1000mz.m	Instrument / Ser#	maXis 29
Sample Name	MB-23_pos		
Comment			

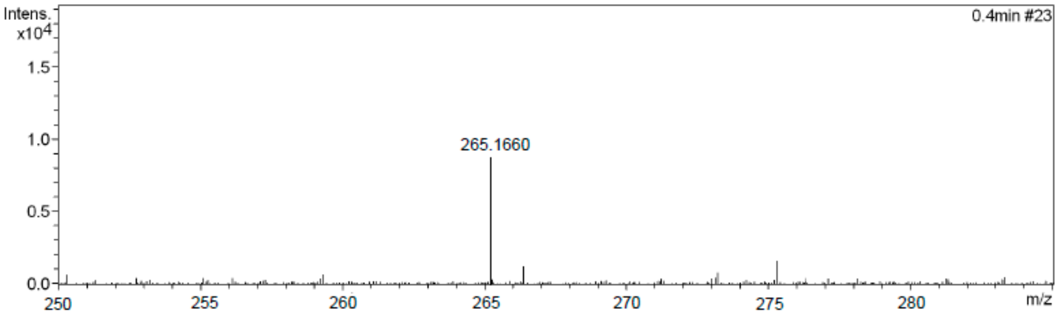

Meas. m/z	#	Relative Intensity	Theoretical Mass	err [ppm]	err [mDa]	Composition
265.1660	1	100.00	265.1678	-6.8	-1.8	C 15 H 23 N O 3

Figure S7. HRESIMS spectrum of compound **1**.

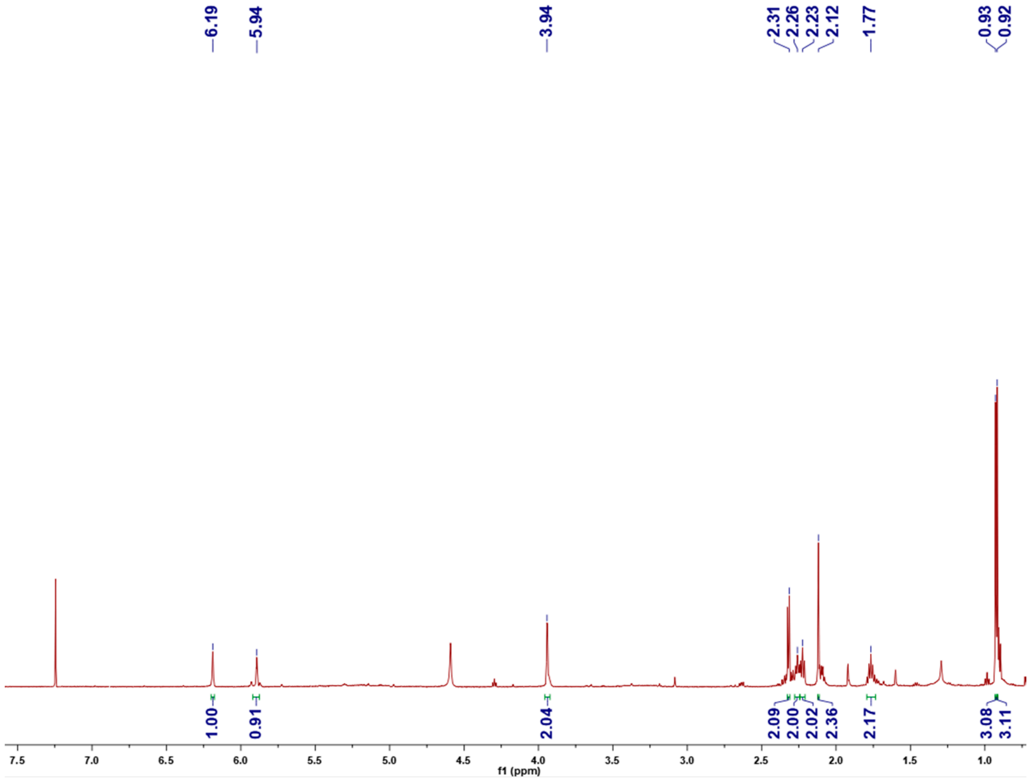

Figure S8. ^{1}H NMR spectrum of compound **2** in $CDCl_3$.

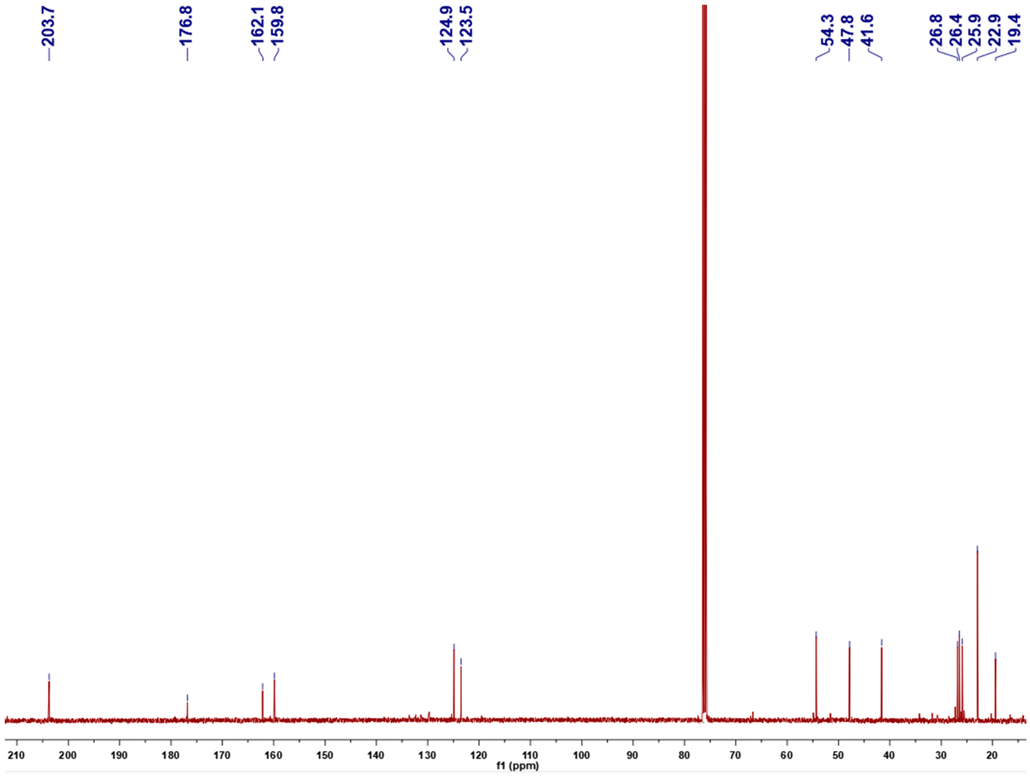

Figure S9. ^{13}C NMR spectrum of compound **2** in $CDCl_3$.

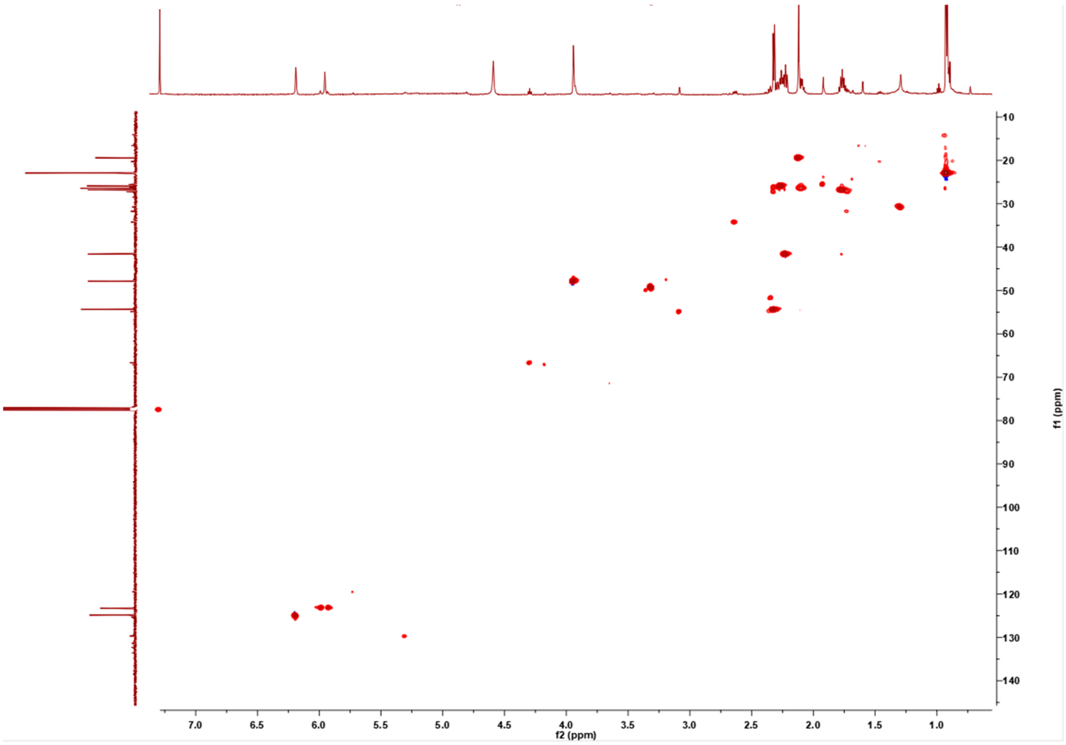

Figure S10. HSQC spectrum of compound **2** in $CDCl_3$.

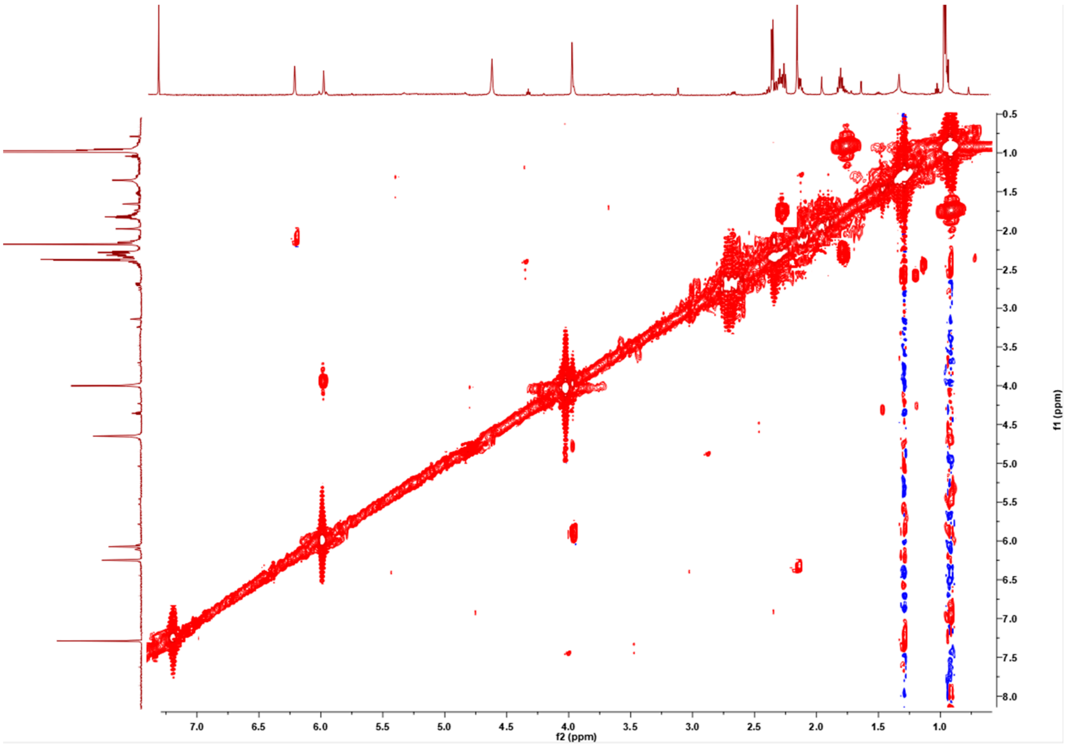

Figure S11. ^{1}H-^{1}H COSY spectrum of compound **2** in $CDCl_3$.

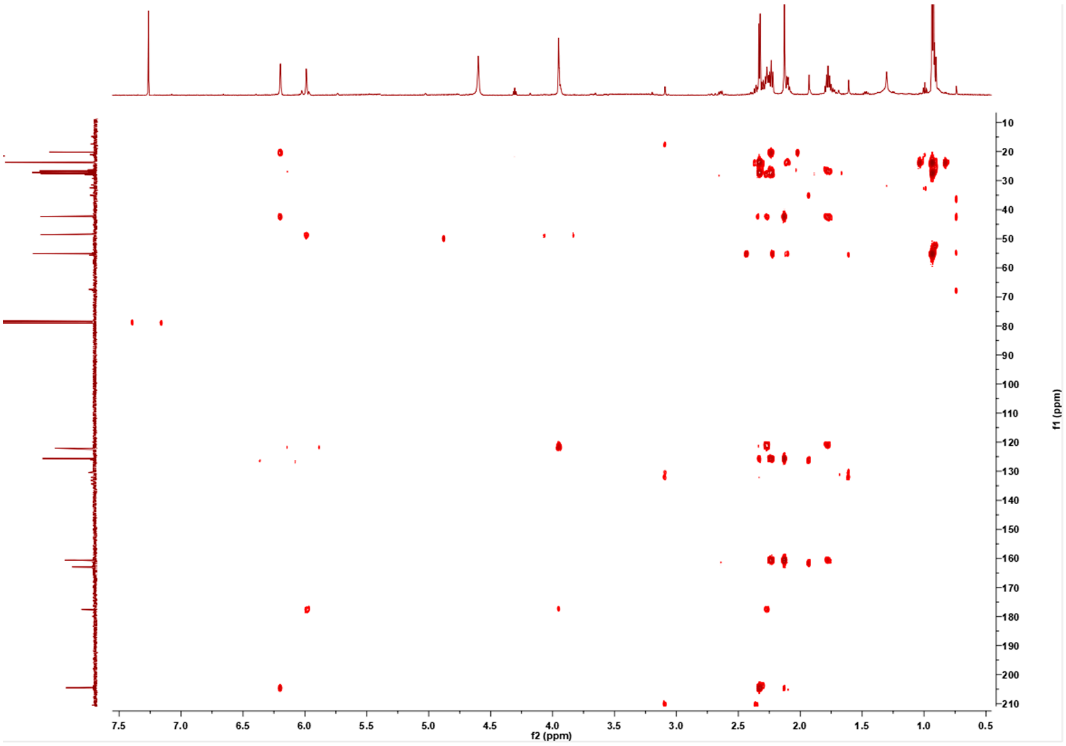

Figure S12. HMBC spectrum of compound **2** in $CDCl_3$.

Mass Spectrum SmartFormula Report

Analysis Info

Analysis Name	D:\Data\MS\data\MB-6_pos.d	Operator	SCSIO
Method	LC_Direct Infusion_pos_100-1000mz.m	Instrument / Ser#	maXis 29
Sample Name	MB-6_pos		
Comment			

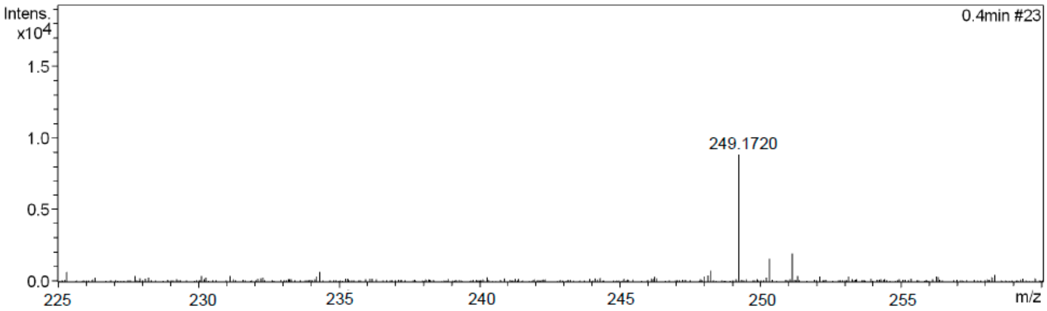

Meas. m/z	#	Relative Intensity	Theoretical Mass	err [ppm]	err [mDa]	Composition
249.1720	1	100.00	249.1723	-1.3	-0.3	C 15 H 23 N O 2

Figure S13. HRESIMS spectrum of compound **2**.
